# Re-association of Body Parts: Illusory Ownership of a Virtual Arm Associated With the Contralateral Real Finger by Visuo-Motor Synchrony

**DOI:** 10.3389/frobt.2020.00026

**Published:** 2020-03-19

**Authors:** Ryota Kondo, Yamato Tani, Maki Sugimoto, Kouta Minamizawa, Masahiko Inami, Michiteru Kitazaki

**Affiliations:** ^1^Department of Computer Science and Engineering, Toyohashi University of Technology, Toyohashi, Japan; ^2^Department of Information and Computer Science, Keio University, Yokohama, Japan; ^3^Graduate School of Media Design, Keio University, Yokohama, Japan; ^4^Research Center for Advanced Science and Technology, The University of Tokyo, Bunkyo-ku, Japan

**Keywords:** illusory body ownership, sense of agency, re-association, visuo-motor synchrony, virtual reality

## Abstract

Illusory ownership can be induced in a virtual body by visuo-motor synchrony. Our aim was to test the possibility of a re-association of the right thumb with a virtual left arm and express the illusory body ownership of the re-associated arm through a synchronous or asynchronous movement of the body parts through action and vision. Participants felt that their right thumb was the virtual left arm more strongly in the synchronous condition than in the asynchronous one, and the feeling of ownership of the virtual arm was also stronger in the synchronous condition. We did not find a significant difference in the startle responses to a sudden knife appearance to the virtual arm between the two synchrony conditions, as there was no proprioceptive drift of the thumb. These results suggest that a re-association of the right thumb with the virtual left arm could be induced by visuo-motor synchronization; however, it may be weaker than the natural association.

## Introduction

Body ownership can be induced not only in real or realistic bodies but also in fake or virtual bodies. Examples of such illusory body ownership include the rubber hand illusion (Botvinick and Cohen, 1998; Longo et al., 2008). In this illusion, body ownership was induced for a rubber hand by stroking a rubber hand and the observer's hand simultaneously. The observer felt that the rubber hand belonged to his own body. In this example, a visuo-tactile integration induced the illusory body ownership. In other cases, visuo-motor synchrony has been used to induce an illusory body ownership (Gonzalez-Franco et al., 2010; Sanchez-Vives et al., 2010). For example, when a virtual avatar's movement is synchronized with an observer's movement, the observer feels as if the avatar is his own body. The illusory body ownership is stronger in the visuo-motor synchrony method than in the passive visuo-tactile integration method (Kokkinara and Slater, 2014).

Induction of the rubber hand illusion needs many preconditions: the rubber hand and the observer's hand must be in the same posture (Ehrsson et al., 2004; Tsakiris and Haggard, 2005), and synchronous stimuli need to be provided for ~23 s (Kalckert and Ehrsson, 2017). The illusory ownership can also be induced with a bright light from a laser pointer without any touch stimulus, and the tactile and thermal sensations can be provided simultaneously (Durgin et al., 2007). The illusion has been expanded to two rubber hands (Ehrsson, 2009), and further to a third arm illusion, with a rubber hand and the visible own hands by visuo-tactile integration (Guterstam et al., 2011).

Illusory body ownership may be induced for a body in a different color to decrease implicit racial bias (Maister et al., 2013; Peck et al., 2013). Similarly, it may be induced as a child avatar for adult observers; then, the illusion can modulate implicit attitudes and object-size perception (Banakou et al., 2013). The ownership can be induced to an empty space like an invisible body (Guterstam et al., 2013, 2015; van der Hoort and Ehrsson, 2016; Kondo et al., 2018). Thus, an illusory body ownership can be induced, even for a different age, race, or visibility, through visuo-tactile integration or visuo-motor synchrony. These studies focused only on body appearance, shape, posture, and position. The studies have hardly reported on the difference in correspondence between the actual and virtual/illusory body parts—the correspondence has been mostly of natural association. For example, only the right hands of the rubber and the observer are stroked by a brush simultaneously in the rubber hand illusion. In an active method, when observers move their right arm, the avatars' right arm moves.

On the other hand, Sasaki et al. (2017) created a four-arm interaction by adding two robot arms to a body and synchronized them with left and right foot movements. However, the sense of ownership has not been investigated with the robotic system. In Won et al. (2015), participants controlled the avatar's arms using their legs in the virtual environment, and they could quickly learn to control the avatar. As a related work, Petkova and Ehrsson (2009) indicated that an illusory touch can be induced in the right rubber hand when it is brushed simultaneously and synchronously with the observer's left hand. However, the illusion occurred only when a homologous pair of hands was brushed. In contrast, the rubber hand illusion was eliminated when an experimenter touched different fingers between participants' hand and the rubber hand (Kammers et al., 2009). Although these studies manipulated the correspondence between body parts, it has not been clarified whether the illusory body ownership can be induced by body-part re-association at different levels of the human body hierarchy.

In phantom limb studies, a reorganization of the primary somatosensory area (S1) has been reported (Ramachandran et al., 1992; Yang et al., 1994; Ramachandran and Altschuler, 2009). Patients feel touch in the amputated arm when the face is touched (Ramachandran et al., 1992). This result suggests that the map of S1 was rewritten, because there was no signal on the hand region owing to amputation. Therefore, the face region sensations seem to have spread to the hand region. In a study with magnetoencephalography, the hand area was activated when the face was touched (Yang et al., 1994), thus demonstrating that S1 remapping occurs for amputee patients.

In this study, we aimed to see whether a body-part re-association is induced by visuo-motor synchrony in healthy adults. We focused on the re-association of the real right thumb and a virtual left arm because although the right thumb and the left arm are at different levels of the human body hierarchy, the directions of their movements are similar.

## Materials and Methods

### Participants

Twenty volunteers participated in the experiment [all male and right-handed, mean 23.2 years old ± 2.1 standard deviation (SD)]. They had healthy vision and exercise capacity. All participants gave written informed consent before the experiment. All the experiments were approved by the Ethical Committee for Human-Subject Research at Toyohashi University of Technology and were performed in accordance with this committee's guidelines and regulations. The participants were paid for the experiment.

### Apparatus

**Figure 1:**
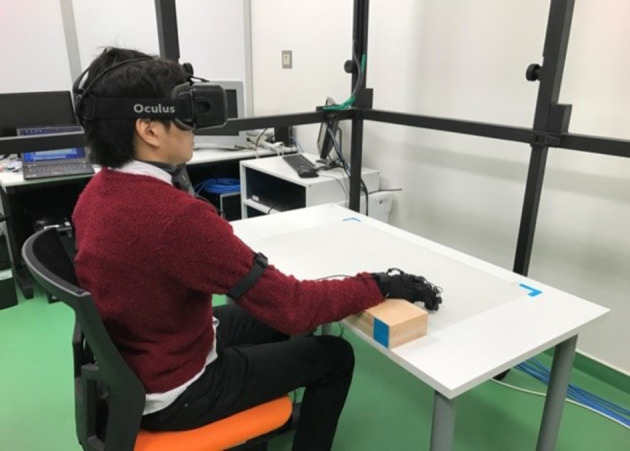
Apparatus.

The participants received stimuli through a head-mounted display (Oculus Rift DK2, 960 (width) × 1,080 (height) pixel, 90 × 110 deg, refresh at 75 Hz). A motion capture system (Noitom Perception Neuron, 120 Hz) detected the observers' right thumb movement. A computer (OS: Windows 10, RAM: 16.0 GB, CPU: Intel(R) Core(TM) i5-6400 CPU @ 2.70 GHz (4 CPUs), GPU: GeForce GTX 1,080) controlled the stimuli. BIOPAC Systems MP150 measured the observers' skin conductance response (SCR) for a threatening stimulus. Two electrodes (EL507) were attached to the distal phalanges of the middle and ring fingers of the participants' left hand. A wireless transmitter (BN-PPGED) was attached to the participants' left wrist, and two lead wires (BN-EDA-LEAD2) were connected from the transmitter to the electrodes. The data were acquired by manufacturer's software AcqKnowledge 4.4 for Windows at a sample rate of 1,000 Hz. The computer sent a trigger signal to an interface module (UIM 100c) connected to the MP150 via Arduino Uno. The trigger was set 10 s before the threatening stimulus, and the data were acquired for 20 s. The participants' right hand was put on a wrist rest so that they could move the thumb freely with the hand facing down (Figure 1). They put their real left arm down at their side.

### Stimuli and Conditions

**Figure 2:**
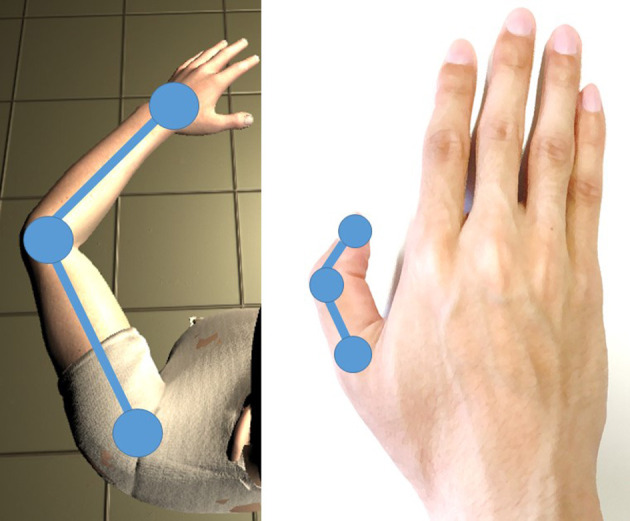
Joint correspondence of the thumb and the virtual arm.

**Figure 3:**
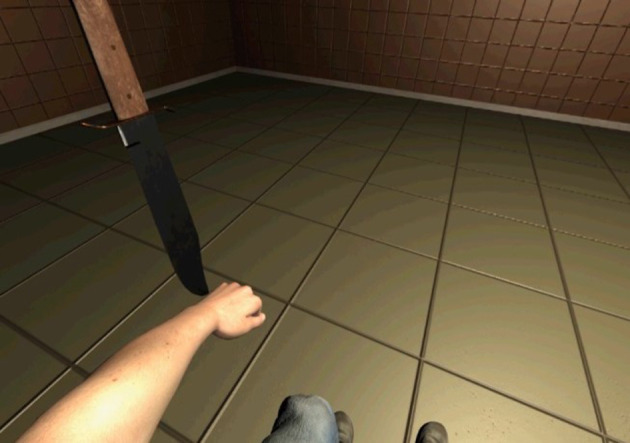
A virtual left arm and a virtual knife.

We presented a virtual left arm that moved synchronously or asynchronously with the observers' right thumb action. The tip and joints of the thumb were associated with the joints of the arm in the synchronous condition; their positions were used to move the virtual arm (Figure 2). The tip of the right thumb was associated with the wrist of the virtual left arm, the proximal interphalangeal joint of the right thumb was associated with elbow joint of the virtual left arm, and the metacarpophalangeal joint of the right thumb was associated with the shoulder of the virtual left arm. We used an inverse kinetics library (Rootmotion Final IK) to generate the movement of the virtual arm from positions of the wrist and the elbow. The postures of the virtual hand and fingers were constant. The participants could see the entire virtual body from a first-person perspective, but they were instructed to observe the virtual left arm. The virtual right hand was not yoked with the actual right hand; it was actually out of sight. One out of two prerecorded motions was selected randomly and presented in the asynchronous condition. A virtual knife appeared to threaten the participants for measuring SCR at the end of each trial (Figure 3). A demonstration video of stimuli is provided as Supplementary Material.

### Procedure

**Figure 4:**
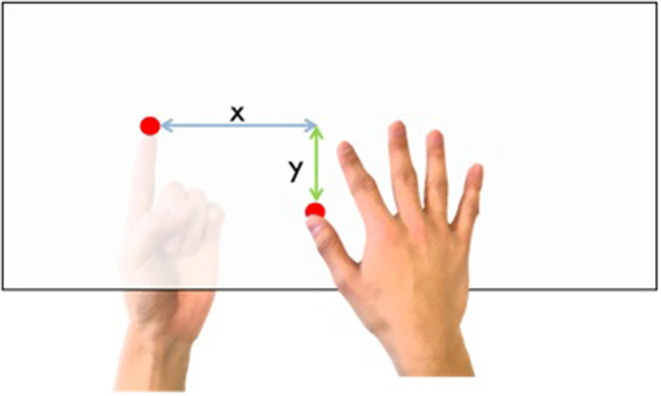
Self-localization task.

Participants observed the virtual left arm while they moved their right thumb freely for 5 min (re-association). Then, a virtual knife appeared on the left arm for measuring SCR as a startle response. Under the table, the participants pointed with their left index finger where they felt the tip of their right thumb (self-localization task, Figure 4). During the localization task, they had the head-mounted display attached and were in the dark. Then, the participants were asked to answer a questionnaire about embodiment on a seven-level Likert scale from 1 (I did not feel it at all) to 7 (I felt it extremely strongly).

1. It felt as if my right thumb became the left arm that I saw.
2. It felt as if the left arm I saw was my left arm.
3. It felt as if my right thumb drifted toward the left arm that I saw.
4. It felt as if my right thumb became longer.
5. It felt as if my left arm increased to two.
6. It felt as if my right thumb became my left thumb.
7. It felt as if the movements of the left arm I saw were my own movements.
8. It felt as if the movements of the left arm I saw were another's movements.

Each participant performed four experimental trials (2 conditions × 2 repetitions) in SAAS (S: Synchronous condition, A: Asynchronous condition) and ASSA order. Two control trials were performed (no re-association period) before the first and after the final trial. In the control trials, the participants were asked to move their right thumb without the virtual body for 5 min, and then they perform the self-localization task (pointing the location of their right thumb with their left index finger without vision) without re-association period. This allowed to calibrate the self-localization measurements of each participant. We subtracted the mean of both control trials from the measured data of the test trials.

## Results

Wilcoxon signed-rank test was used for a statistical test of the results of the questionnaire. The probability of superiority of dependent measurements (PS_dep_) showed the effect size. Paired *t*-tests were applied to test the difference between the synchronous and asynchronous conditions using the SCR data from the startle response, the data of self-localization drift, and the length of the finger the participants perceived. Cohen's d showed the effect size. We hypothesized that the illusory re-associated body ownership of the virtual left arm should occur more in the synchronous condition than in the asynchronous one. Data of all participants are provided as Supplementary Material.

### Questionnaire

**Figure 5:**
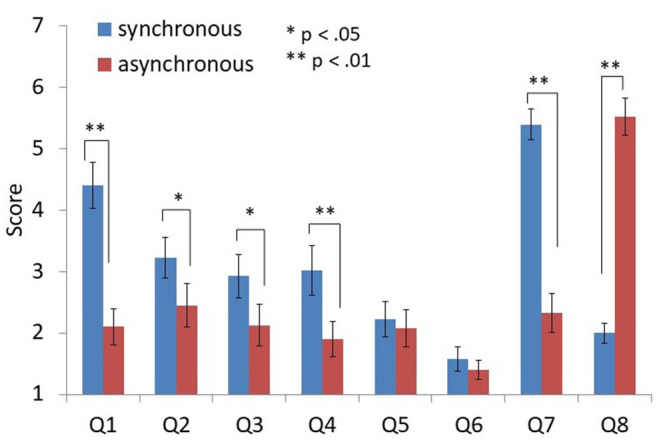
Results of the questionnaire. The error bars indicate standard errors (SE). *, ** indicate statistical significance at the 0.05 (*p* < 0.05) and 0.01 (*p* < 0.01) levels with Wilcoxon signed-rank test, respectively.

The results of the questionnaire are shown in Figure 5. Participants felt that their right thumb was the left arm (Q1) more strongly in the synchronous condition than in the asynchronous condition [*z* (19) = 3.63, *p* < 0.01, PS_dep_ = 0.85]. They also felt that the virtual left arm was their left arm (Q2) more strongly in the synchronous condition [*z* (19) = 2.16, *p* = 0.031, PS_dep_ = 0.70]. They felt that their right thumb drifted toward the left arm (Q3) more strongly in the synchronous condition than the asynchronous condition [*z* (19) = 2.40, *p* = 0.014, PSdep = 0.60]. The feeling of their right thumb growing longer (Q4) was also more prevalent in the synchronous condition than in the asynchronous one [*z* (19) = 2.78, *p* < 0.01, PS_dep_ = 0.60]. Furthermore, the participants felt the movement of their left arm as their own (Q7) more strongly in the synchronous condition [*z* (19) = 3.84, *p* < 0.01, PS_dep_ = 0.95].

By contrast, they felt the movements of their left arm as another's movements (Q8) more strongly in the asynchronous condition than in the synchronous condition [*z* (19) = −3.93; *p* < 0.01, PS_dep_ = 1.00].

There was no significant difference in the other items [Q5: *z* (19) = 0.56, *p* = 0.63, PS_dep_ = 0.35; Q6: *z* (19) = 1.33, *p* = 0.25, PS_dep_ = 0.3]. They did not feel the left arm increasing to two (two left arms), suggesting that the virtual left arm did not become the second left arm or the third arm (Q5). Additionally, the participants did not feel that the right thumb became the left thumb (Q6). Actually, Q6 was a control question to check possible participants' random (unreliable) responses.

### Startle Response

**Figure 6:**
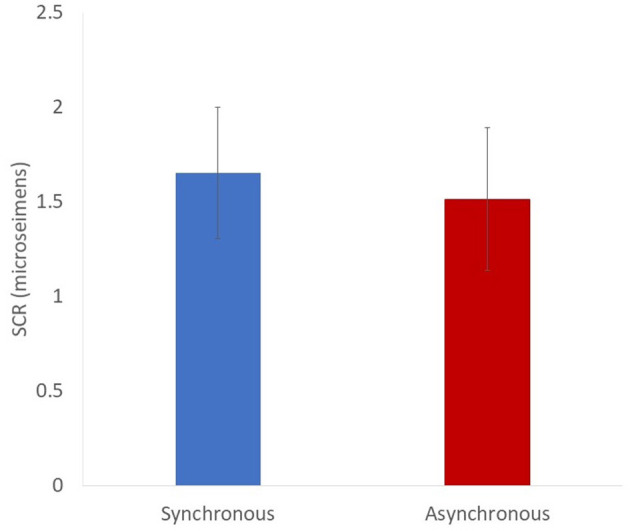
Result of startle response (mean). The error bars indicate SE.

The amplitude of SCR was calculated as the difference between the maximum and minimum level of skin conductance in the period of 0–5 s after the knife appearance. It was based on previous studies (Petkova and Ehrsson, 2008; Guterstam et al., 2011, 2013, 2015; Petkova et al., 2011; Guterstam and Ehrsson, 2012). We did not find a significant difference in SCRs between the synchronous and asynchronous conditions [Figure 6, *t* (19) = 0.62, *p* = 0.54, *d* = 0.09].

### Self-localization Task

We hypothesized that if the observers felt that the right thumb became the left arm, then the proprioceptive location of the right thumb would drift toward the left arm, and the thumb would be perceived as being longer than its actual length. We defined the position in the *x* (horizontal) direction as the self-localization drift and the position in *y* (vertical) direction as the length of the finger perceived by them. The average of the control trials was subtracted from that of the experimental trials to control the participants' individual differences.

**Figure 7:**
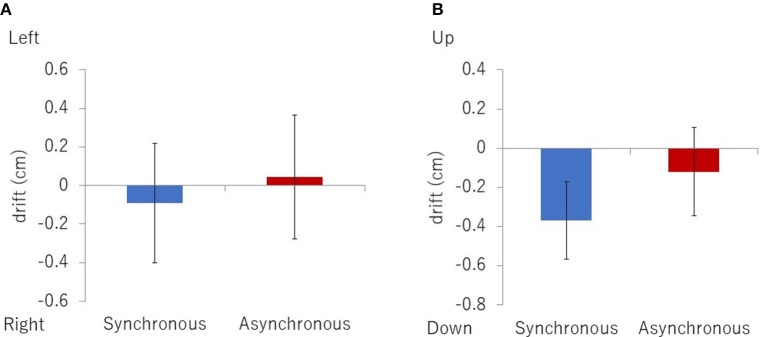
Results of self-localization drift **(A)** and perceived length of the finger **(B)**.

There was no significant difference between the synchronous and asynchronous conditions for the self-localization drift (x) [Figure 7A, *t* (19) = −0.39, *p* = 0.70, *d* = 0.10] as well as for the length of the finger perceived (y) [Figure 7B, *t* (19) = −1.37, *p* = 0.19, *d* = 0.27].

## Discussion

We found that the participants felt as if their own right thumb had become the left arm and illusory body ownership of the virtual left arm was induced more in the visuo-motor synchronous condition than in the asynchronous one (illusory re-associated ownership). The SCR for the threatening stimulus to the left arm did not show a significant difference between the synchronous and asynchronous conditions. The self-localization drift or the elongation of the right thumb by the illusory re-associated ownership of the left arm did not occur in the behavioral task.

The results of the questionnaire suggest that the synchrony of the real right thumb action and the movement of the virtual left arm could induce an illusory re-associated ownership of the left arm. However, the scores of re-association and ownership are not high [Q1: mean 4.4; Q2: mean 3.2 (1-to-7 Likert scale); both in the synchronous condition]. The SCR (startle response) and the behavioral data of the self-localization task did not support our hypothesis. Thus, the illusory re-associated ownership can definitely be induced in the current method, but only weakly. We speculate that the natural association (such as the real left arm and the virtual left arm) conflicts with the virtual re-association, and it diminishes the effect of the visuo-motor synchrony. In previous studies of brain remapping by neural plasticity, patients have lost their natural associations with amputation (Ramachandran et al., 1992; Aglioti et al., 1994, 1997; Yang et al., 1994) or brain damage (Clarke et al., 1996; Turton and Butler, 2001). It is an open question whether the re-association or dual association of body parts in the brain could be induced with the natural association intact. Our method might become a good tool for investigating such neural plasticity.

Kilteni et al. (2012) defined the sense of embodiment as the sense that emerges when one's own body's properties are processed as if they were the properties of one's own biological body, and they proposed that it comprises three subcomponents: the senses of self-location, agency, and body ownership. In our results, the subjective measures corresponding to these three subcomponents were significantly higher in the synchronous condition than in the asynchronous condition. From the questionnaire, we found significant differences between the synchronous and asynchronous conditions in terms of feelings of illusory body ownership, body-part re-association, the finger's subjective drift and elongation, and a sense of agency, although we did not observe differences in terms of SCR or self-localization tasks. On the other hand, Blanke and Metzinger (2009) proposed the minimal phenomenal selfhood (MPS), which is related to the concept of embodiment and the conscious experience of being a self; it is also characterized by the ownership of a whole body, self-location, and the first-person perspective. In contrast to MPS, our re-association body ownership is limited to body-part ownership, and hardly disturbs MPS in terms of global body ownership (especially of the trunk and head), global self-location, and the first-person perspective.

However, the participants' responses on body ownership might be affected by the perceived sense of agency. The sense of body ownership and the sense of agency mutually strengthen each other unless the experimental method prevents their concomitant emergence (Kalckert and Ehrsson, 2012; Braun et al., 2018); Zopf et al. (2018) demonstrated that the motion congruency of the participant's hand and an object (sphere) increased the sense of ownership as well as the sense of agency. In the present study, the participants moved their right thumb voluntarily and the virtual left arm moved congruently so that the sense of ownership could not be dissociated from the sense of agency and might be enhanced by the voluntary motion congruency.

The scores from the questionnaire of illusory body ownership (like “I felt as if the rubber hand was my hand”) for the visuo-motor rubber hand illusion are usually around 2 (−3 to +3 Likert scale) in previous studies (e.g., Ehrsson, 2007; Kalckert and Ehrsson, 2014a,b). Thus, our score (4.4 in 1-to-7 Likert scale) is much lower than that of natural association in the previous studies, although there is a significant difference between the synchronous and asynchronous conditions. This is a limitation of our study that should be addressed in the future. We would like to add tactile stimuli to the re-association setup to increase the sense of body ownership in a future study.

In the present study, participants moved their right thumb freely and observed the virtual left arm for 5 min in the experiment, similar to previous studies. For example, the studies on the illusory ownership of a hand (Sanchez-Vives et al., 2010) and a full body (Gonzalez-Franco et al., 2010) exposed participants to stimuli for 3 min, whereas the study of the rubber hand illusion on the contralateral hand had 5 min as the exposure time (Petkova and Ehrsson, 2009). However, a longer exposure time might improve the illusory ownership of the re-associated body parts.

In heautoscopy, patients often experience two different bodies at two distinct spatial locations (Brugger et al., 2006). However, out-of-body illusion studies have shown that body ownership cannot be induced with two full bodies of the physical body and the illusory body simultaneously (Ehrsson, 2007; Guterstam and Ehrsson, 2012). In an experiment, a participant observed video camera images of his or her back through an HMD, and illusory body ownership was induced in a space in front of a camera by touching simultaneously the participant's actual chest and a space in front of the camera. Additionally, the body ownership of their actual body decreased (Guterstam and Ehrsson, 2012). On the contrary, when the participants observed two fake hands (two right rubber hands (Ehrsson, 2009) or a right rubber hand and observer's right hand in sight Guterstam et al., 2011), they assumed an illusory ownership of the two hands by visuo-tactile stimulation. For full body illusion, we have self-identification with two virtual bodies or two physical (video-image) bodies when two virtual bodies are presented side by side in front of us and their backs as well as our physical back are stroked synchronously (Heydrich et al., 2013). Hence, illusory ownership can be induced in two similar fake body parts and two similar virtual/pictorial full bodies. These suggest that there is a limitation or rule of illusory body change or editing. Our aim was to induce re-association of different body parts at different hierarchies, such as the finger and the arm. We speculate that an analogous structure and movement are necessary for the re-association to elicit the appropriate sense of agency, and hence contribute to the illusory ownership. Therefore, the joints of the thumb were mapped to the joints of the arm, which can be done because the structure and movement between the finger and the arm are similar. Additionally, we used both the right thumb and the virtual left arm (contralateral combination) in a hand-face-down posture and set their motion directions similarly. However, the question is still open as to how much two body parts must be analogous for re-association. It must be further investigated in a future study.

The concept of re-association of body parts may contribute to developing functional prostheses. Body-powered prostheses are more durable and require less training, but their appearance attracts attention and their functions are limited; myoelectric prostheses are more expensive and require more training, but their appearance is natural and their functions can be improved by signal processing of EMG and training (Antfolk et al., 2013; Carey et al., 2015). Based on our study, we propose a modified system of body-powered prosthesis with electrical power controlled by body movements. If an upper-limb prosthesis is controlled by a different body part such as the finger of an undamaged hand, people can use it with less training and control it more naturally than conventional prostheses. In the present experiment with healthy participants, the sense of the actual left arm might be conflicted with the illusory ownership of the virtual left arm. For amputees, this conflict is irrelevant, and we speculate that learning will require less time and body ownership will occur more for them than for the healthy participants. This speculation is partly supported by the following neurological findings in amputee patients, as discussed in previous literature. Reorganization of the primary somatosensory area occurs in phantom limb patients (Ramachandran et al., 1992; Yang et al., 1994; Ramachandran and Altschuler, 2009). The use of a myoelectric prosthesis prevents cortical reorganization and phantom limb pain (Lotze et al., 1999), and the upper limb amputees exhibit significantly higher activation in the contralateral primary motor and somatosensory cortices while imagining moving the phantom hand, compared with the imagination of hand movements in the healthy participants (Lotze et al., 2001). The rubber hand illusion can be induced in upper limb amputees and is associated with activity in the premotor and the intraparietal cortices (Schmalzl et al., 2014). These suggest that the cortical area corresponding to the amputated limb has the potential to control and sense the re-associated body part if it represents the amputated body part. Thus, it is important to further study the sense of body ownership and agency of re-associated body parts for potential future application to prostheses.

## Data Availability Statement

All datasets generated for this study are included in the article/Supplementary Material.

## Ethics Statement

The studies involving human participants were reviewed and approved by The Ethical Committee for Human-Subject Research at Toyohashi University of Technology. The patients/participants provided their written informed consent to participate in this study.

## Author Contributions

MK, RK, YT, MS, KM, and MI conceived and designed the experiments. RK and YT collected and analyzed the data. RK and MK contributed to the preparation of the manuscript. All images, drawings, and photographs were obtained or created by RK. All authors reviewed the manuscript.

### Conflict of Interest

The authors declare that the research was conducted in the absence of any commercial or financial relationships that could be construed as a potential conflict of interest.

## Supplementary Material

Supplementary Table 1 Experiment Data.

Supplementary Video 1 Demonstration Video.
